# Systemic Immune-Inflammation Index as a Predictor of Progression in Melanoma: A Retrospective Cohort Study

**DOI:** 10.7759/cureus.94939

**Published:** 2025-10-19

**Authors:** Ana G Perez-Romero, Martha A Aceves Villalvazo, Arcelia Y Figueroa Martínez, Monica P Ramos Alvarez

**Affiliations:** 1 Dermatology, Hospital Regional "Dr. Valentín Gómez Farías" Institute for Social Security and Services for State Workers (ISSSTE), Zapopan, MEX; 2 Dermatology, Dermika, Centro Dermatológico Laser, Guadalajara, MEX

**Keywords:** melanoma, prognostic biomarker, progression-free survival, risk stratification, systemic immune-inflammation index (sii)

## Abstract

Background: Systemic inflammation plays a pivotal role in melanoma progression through mechanisms of immune suppression, angiogenesis, and metastatic dissemination. The systemic immune-inflammation index (SII), integrating platelet, neutrophil, and lymphocyte counts, has emerged as a composite biomarker reflecting the balance between tumor-promoting inflammation and host immune response.

Objective: This study aimed to evaluate the prognostic value of the SII in predicting 12-month progression-free survival (PFS) in patients with melanoma and to determine whether elevated SII independently correlates with disease progression after adjustment for clinical stage and age.

Methods: A retrospective cohort study was conducted, including 40 patients with histopathologically confirmed melanoma between 2019 and 2024. Baseline SII was calculated as (platelet count × neutrophil count) / lymphocyte count, using a validated cutoff of 560. Statistical analyses were performed using *Jamovi* version 2.4. Associations between SII and 12-month outcomes were assessed using chi-square and logistic regression analyses, while Kaplan-Meier and ROC analyses evaluated prognostic performance.

Results: An SII ≥560 was significantly associated with higher progression risk (relative risk (RR) 5.3; odds ratio (OR) 22.7, 95% confidence interval (CI) 4.4-117.5; p < 0.001). Patients with elevated SII had markedly reduced 12-month PFS (20% vs. 85%; log-rank = 16.5, p < 0.001). SII showed excellent discriminative ability (area under the curve (AUC) 0.91, 95% CI 0.83-0.99). In multivariable analysis, SII remained independently predictive of progression (adjusted OR 14.6; 95% CI 2.3-91.8; p = 0.004).

Conclusion: The SII independently predicted 12-month disease progression in melanoma, identifying high-risk patients even within early stages. These findings support its role as a simple, reproducible, and low-cost biomarker for preoperative risk assessment and individualized follow-up. Future prospective multicenter studies with larger cohorts and extended follow-up are warranted to validate these results and to better define the clinical utility of SII as part of comprehensive melanoma risk stratification strategies.

## Introduction

Skin cancers rank among the most common malignancies worldwide, with especially high incidence in the United States, Australia, and Europe [1]. Within this spectrum, melanoma, despite representing a smaller fraction than non-melanoma skin cancers, stands out for its marked aggressiveness and metastatic potential, highlighting its importance as a public health concern [1]. Tumor progression in melanoma is closely intertwined with host immunity, where systemic inflammation functions as a key promoter of oncogenesis and dissemination [2].

Circulating inflammatory cells - neutrophils, lymphocytes, and platelets - play a consistent role in tumor biology [2,3]. Mechanistically, neutrophils release proteases, reactive oxygen species, and cytokines that foster angiogenesis, remodel the extracellular matrix, and facilitate the adhesion of circulating melanoma cells at distant sites [3]. Platelets shield tumor cells from immune clearance and aid metastatic seeding through vascular adhesion and growth factor release [4]. Lymphocytes, particularly cytotoxic T cells, support antitumor surveillance by recognizing tumor antigens, inducing apoptosis, and restraining spread [5].

Recent evidence indicates that alterations in other immune cell populations, particularly myeloid and plasmacytoid dendritic cells (mDCs and pDCs) and regulatory T cells (Tregs), also contribute to melanoma progression [6]. High Treg levels and reduced dendritic cell counts have been associated with impaired antitumor immunity and worse prognosis [6]. Tregs inhibit myeloid dendritic cell migration by suppressing CCR7 expression and promoting IL-10 secretion, while reduced mDCs compromise antigen presentation and cytotoxic T-cell activation. Conversely, pDCs, although less sensitive to Treg regulation, tend to decrease in advanced melanoma, further reflecting systemic immune suppression. Together, these findings support the concept that an imbalance between pro-inflammatory and immunoregulatory cells can mirror disease activity and recurrence risk in melanoma [6].

In this context, the systemic immune-inflammation index (SII), calculated as neutrophil count × platelet count/lymphocyte count, integrates pro-tumor inflammatory activity with antitumor immune surveillance capacity [7]. Elevated SII has been associated with an inflammatory tumor microenvironment conducive to proliferation, angiogenesis, and metastasis, and multiple studies, including meta-analyses, have reported its association with worse overall and progression-free survival (PFS) across solid tumors, including melanoma [1,8]. The biological rationale is clear and biologically consistent: higher neutrophil and platelet counts amplify tumor-promoting inflammation, vascular dissemination, and immune evasion, while reduced lymphocytes reflect impaired immune surveillance, yielding a systemic state aligned with invasion, metastasis, and poorer outcomes [9,10]. Based on this rationale, we conducted a retrospective cohort study to evaluate the correlation between SII, using the validated cutoff of 560, and 12-month PFS in melanoma, independent of clinical stage, and to determine whether elevated SII remains independently associated with disease progression after adjustment for age and clinical stage [2].

## Materials and methods

This study was approved by the Ethics Committee of Hospital Regional “Dr. Valentín Gómez Farías”, ISSSTE (protocol approval number ISSSTE/CIEB/770/2025). The requirement for informed consent was waived due to its retrospective design. Patients aged >18 years with a histopathological diagnosis of melanoma managed in outpatient dermatology, surgical oncology, geriatrics, internal medicine, and oncology clinics between 2019 and 2024 were included. Inclusion criteria were biopsy-confirmed diagnosis, a minimum follow-up of 12 months, and availability of baseline neutrophil, platelet, and lymphocyte counts at diagnosis. Exclusion criteria included active infections, history of systemic hematologic disorders, prior neoadjuvant therapy, concomitant primary cancer, or use of anti-inflammatory or immunosuppressive medications.

All eligible patients during the study period were included (n = 40). Based on previous reports evaluating inflammatory indices in melanoma, a minimum of 34 participants was estimated to provide 80% power to detect a large effect size (Cohen’s w = 0.5) for the association between SII and 12-month progression, assuming α = 0.05; therefore, the achieved sample size ensured adequate exploratory power.

Demographic variables (age, sex), clinical variables (disease duration, tumor location), histopathologic characteristics (subtype and stage according to the 8th edition of the American Joint Committee on Cancer), and 12-month outcome variables were collected. The primary endpoint was 12-month PFS, defined as the interval from diagnosis to clinical progression, locoregional recurrence, metastasis, or death, in accordance with RECIST v1.1. Outcomes were categorized as favorable (no event) or unfavorable (progression, recurrence, metastasis, or death). The SII was calculated as (neutrophil count × platelet count) / lymphocyte count, expressed in ×10⁹/L. The cutoff value of 560 was adopted from a previously validated meta-analysis [11] and confirmed internally through receiver operating characteristic (ROC) analysis, which showed optimal sensitivity and specificity for 12-month progression prediction.

Statistical analyses were performed using Jamovi version 2.4 (The Jamovi Project, Sydney, Australia). Measures of central tendency and dispersion were used for continuous variables, and frequencies and proportions for categorical variables. The association between SII and 12-month outcomes was assessed using the Chi-square (χ²) test (χ² = 16.9, df = 1, p < 0.001). PFS was estimated with the Kaplan-Meier method, and survival curves were compared with the log-rank test (log-rank = 16.5, p < 0.001), with p < 0.05 considered significant. An ROC curve was constructed to evaluate the discriminative capacity of SII, calculating the area under the curve (AUC), sensitivity, and specificity at the cutoff of 560. Absolute risks of progression were estimated for each group (SII <560 and SII ≥560), and from these, relative risk (RR) and odds ratio (OR) with 95% confidence intervals (CIs) were calculated to quantify the association strength between elevated SII and disease progression.

To account for potential confounding effects, a multivariable logistic regression was performed with 12-month progression (yes/no) as the dependent variable and SII (≥560 vs. <560) as the main independent predictor. A priori confounders included age (continuous, in years) and clinical stage at diagnosis (AJCC 8th edition; categorical, with stage I as the reference group). To maintain model parsimony given the sample size, sex was evaluated in a sensitivity analysis and retained only if it altered the SII coefficient by ≥10% or improved model fit according to the Akaike Information Criterion (AIC). Linearity of continuous covariates (age) was verified using fractional polynomial testing, and multicollinearity was ruled out with variance inflation factors (VIF <5). Model calibration was evaluated using the Hosmer-Lemeshow goodness-of-fit test, and discrimination was summarized by the area under the ROC curve (AUC). Results were reported as adjusted odds ratios (aOR) with 95% CIs, and a two-sided p < 0.05 was considered statistically significant.

## Results

A total of 40 patients with histopathologically confirmed melanoma were included in the analysis (Table 1). The median age was 55.5 years (IQR 42.0-65.8), with a female-to-male ratio of 1.8:1 (26 women, 14 men). The median time from lesion onset to diagnosis was 25.4 months. Histologic subtypes included nodular melanoma in 42.5% (n = 17), superficial spreading melanoma in 35% (n = 14), acral lentiginous melanoma in 20% (n = 8), and lentigo maligna melanoma in 2.5% (n = 1). Clinical stages at diagnosis were stage I in 45% (n = 18), stage II in 17.5% (n = 7), stage III in 10% (n = 4), and stage IV in 27.5% (n = 11), according to the AJCC 8th edition.

**Table 1:** Baseline clinical and histopathological characteristics of the study population (n = 40) Distribution of demographic, clinical, and histopathological variables of patients with melanoma according to 12-month outcomes. Favorable outcome: no progression, recurrence, or death within 12 months. Unfavorable outcome: progression, recurrence, metastasis, or death. Statistical analyses were performed using Jamovi version 2.4 (The Jamovi Project, Sydney, Australia). Variables; Mann–Whitney U for continuous variables.

Variable	Total (n = 40)	Favorable outcome (n = 20)	Unfavorable outcome (n = 20)	p-value
Age, median (IQR)	55.5 (42.0–65.75)	51.0 (39.0–60.0)	60.5 (47.0–68.0)	0.12
Sex, n (%): Female / Male	26 (65) / 14 (35)	15 (75) / 5 (25)	11 (55) / 9 (45)	0.18
Time from lesion onset to diagnosis (months), median (IQR)	25.4 (14–38)	22.0 (12–35)	28.0 (18–42)	0.21
Histologic subtype, n (%):				
Nodular	17 (42.5)	6 (30)	11 (55)	0.09
Superficial spreading	14 (35)	9 (45)	5 (25)	
Acral lentiginous	8 (20)	4 (20)	4 (20)	
Lentigo maligna	1 (2.5)	1 (5)	0 (0)	
Clinical stage (AJCC 8th), n (%):				
Stage I	18 (45)	14 (70)	4 (20)	<0.01*
Stage II	7 (17.5)	3 (15)	4 (20)	
Stage III	4 (10)	1 (5)	3 (15)	
Stage IV	11 (27.5)	2 (10)	9 (45)	
SII, n (%):				
<560	19 (47.5)	16 (80)	3 (15)	<0.001*
≥560	21 (52.5)	4 (20)	17 (85)	
Progression at 12 months, n (%):	20 (50)	—	—	—

**Figure 1:**
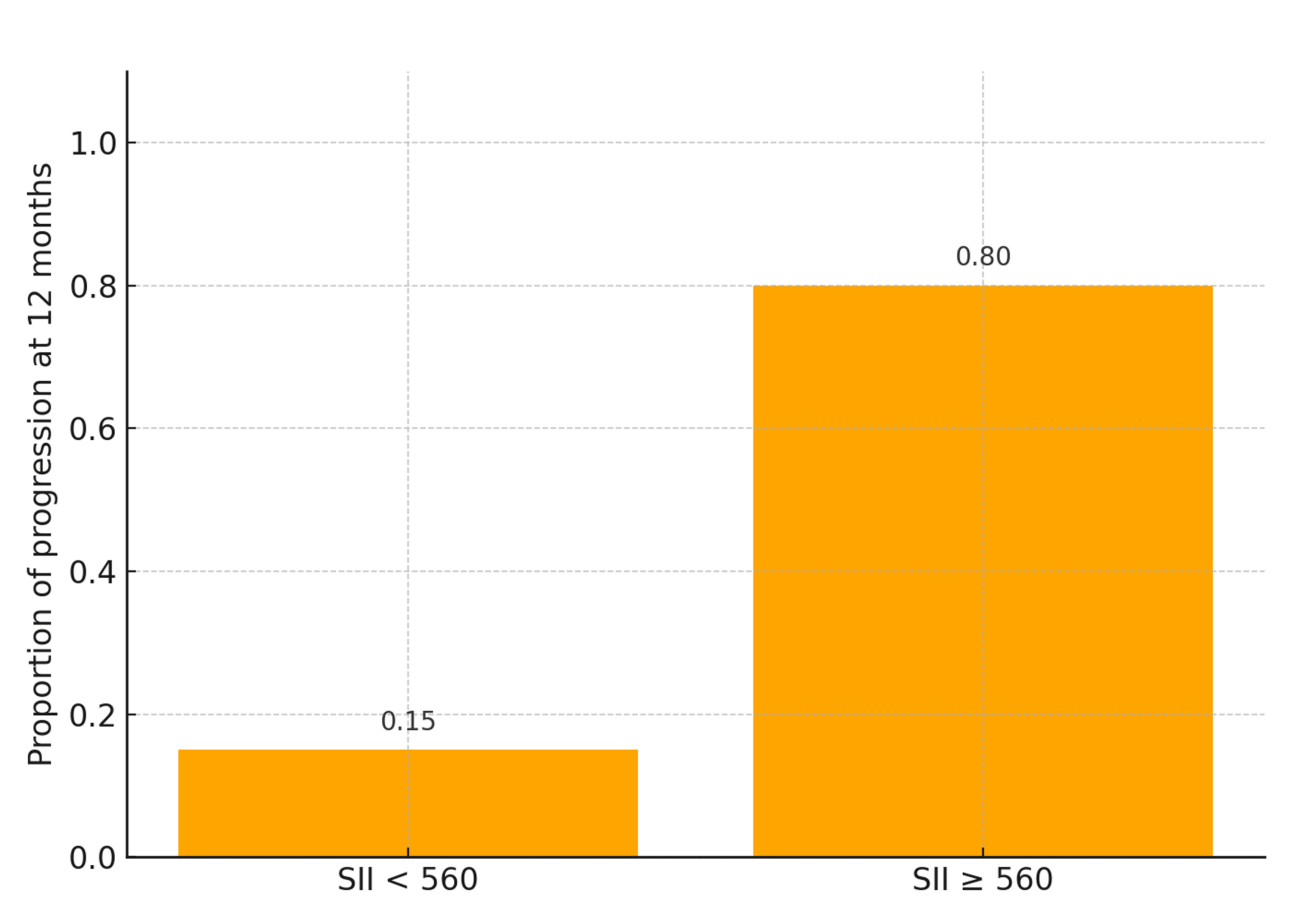
Absolute risk of disease progression at 12 months according to the systemic immune-inflammation index (SII). Patients with SII ≥560 had an 80% risk of progression compared with 15% in those with SII <560 (χ² = 16.9, p < 0.001). Bars represent absolute proportions of unfavorable outcomes at 12 months.

All statistical analyses were performed using Jamovi version 2.4. Outcomes at 12 months were categorized as favorable (no progression, recurrence, or death) or unfavorable (progression, recurrence, metastasis, or death) to allow binary comparison across clinical stages and SII strata. Nineteen patients (47.5%) experienced an unfavorable outcome within 12 months. The comparison between SII <560 and SII ≥560 demonstrated a significant association with 12-month outcomes (χ² = 16.9, df = 1, p < 0.001). Patients with elevated SII had markedly higher progression risk (80%) than those with low SII (15%), corresponding to a relative risk of 5.3 and an odds ratio of 22.7 (95% CI, 4.4-117.5; p < 0.001) (Figure 1).

**Figure 2:**
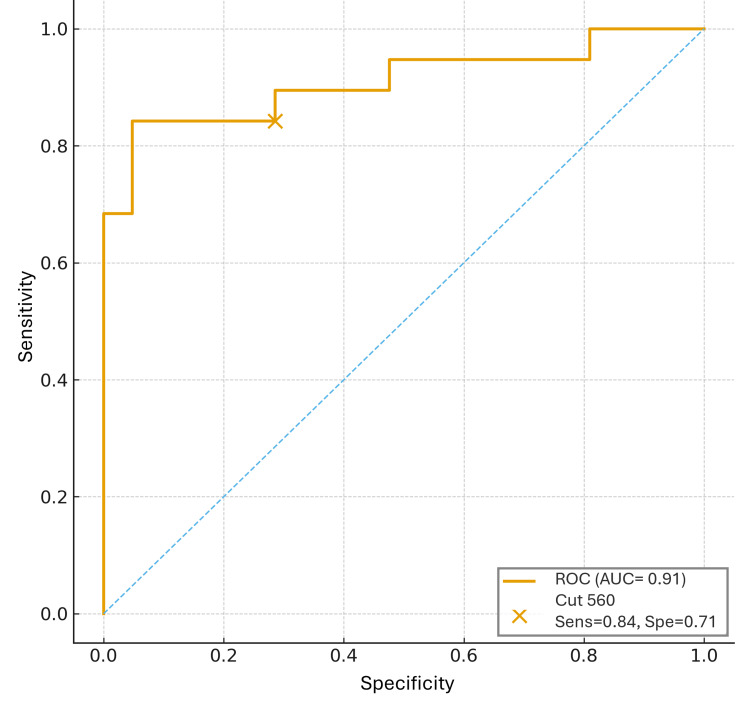
Receiver operating characteristic (ROC) curve of the systemic immune-inflammation index (SII) for predicting 12-month progression in melanoma The area under the curve (AUC) was 0.91 (95 % CI, 0.83–0.99), indicating excellent discriminative capacity. The validated cutoff of 560 yielded 84.2% sensitivity and 71.4% specificity. Statistical analysis and curve generation were performed using Jamovi version 2.4 (The Jamovi Project, Sydney, Australia).

**Figure 3:**
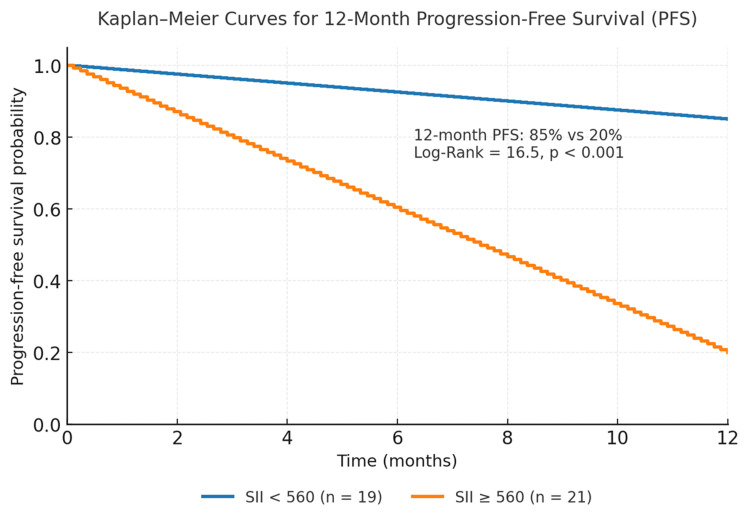
Kaplan–Meier curves for 12-month progression-free survival (PFS) in patients with melanoma stratified by the systemic immune-inflammation index (SII). Patients with SII <560 showed significantly higher PFS (85 %) compared with those with SII ≥560 (20 %) (Log-Rank = 16.5, p < 0.001). Median PFS was not reached in the low-SII group and was 4.8 months in the high-SII group. Survival analysis was conducted using Jamovi version 2.4 (The Jamovi Project, Sydney, Australia).

The ROC curve for SII yielded an AUC of 0.91 (95% CI, 0.83-0.99), confirming excellent discriminative capacity for predicting 12-month progression (Figure 2). Using the 560 cutoff, sensitivity was 84.2% and specificity was 71.4%. Kaplan-Meier analysis demonstrated clear separation of survival curves between both SII groups (log-rank = 16.5, p < 0.001). Median PFS was not reached for patients with SII <560, whereas the SII ≥560 group showed a median PFS of 4.8 months (Figure 3). The estimated 12-month PFS was 85% in the low-SII group and 20% in the high-SII group. Notably, all early-stage cases (I-II) that progressed within 12 months had elevated SII at diagnosis, suggesting that increased systemic inflammation may identify biologically aggressive tumors even in clinically localized disease.

In the multivariable logistic regression model adjusted for age and clinical stage (Table 2), elevated SII remained independently associated with disease progression (adjusted OR = 14.6; 95% CI, 2.3-91.8; p = 0.004). Model calibration was satisfactory (Hosmer-Lemeshow p = 0.72), and the overall discriminative performance remained high (AUC = 0.91). Neither age nor stage significantly modified the association, confirming that SII independently predicts progression risk in melanoma.

**Table 2:** Multivariable logistic regression model for predictors of 12-month disease progression in melanoma. Adjusted model including systemic immune-inflammation index (SII), age, and clinical stage (AJCC 8th edition). Elevated SII (≥560) remained independently associated with disease progression (adjusted OR = 14.6; 95% CI, 2.3–91.8; p = 0.004). Model calibration: Hosmer–Lemeshow p = 0.72; discriminative ability: AUC = 0.91. Analyses were performed using Jamovi version 2.4 (The Jamovi Project, Sydney, Australia). Model performance: Hosmer–Lemeshow test: p = 0.72. AUC (discrimination): 0.91. Statistically significant at p < 0.05.

Variable	Adjusted OR (aOR)	95% CI	p-value
Systemic Immune-Inflammation Index (≥560)	14.6	2.3–91.8	0.004*
Age (per year)	1.03	0.98–1.09	0.22
Clinical stage (ref: Stage I)			
• Stage II	1.8	0.3–9.9	0.51
• Stage III	3.9	0.6–24.4	0.15
• Stage IV	7.1	1.2–42.3	0.03*

## Discussion

Skin cancers are among the most prevalent malignancies worldwide [1]. Melanoma, although less frequent than non-melanoma skin cancers, is characterized by marked aggressiveness and metastatic potential, making it a major public health concern [1]. Chronic inflammation is a recognized driver of tumor initiation, progression, and metastasis [8]. Peripheral inflammatory cells-neutrophils, lymphocytes, and platelets-play key roles in the tumor microenvironment [8,9]. Neutrophils promote tumor growth through the release of proteases, reactive oxygen species, and cytokines that stimulate angiogenesis, remodel the extracellular matrix, and facilitate adhesion of circulating melanoma cells at distant sites [9]. Platelets further contribute by shielding tumor cells from immune surveillance and promoting vascular adhesion and metastatic spread through growth factor release [10]. In contrast, lymphocytes, particularly cytotoxic T cells, mediate immune surveillance by inducing apoptosis of malignant cells and limiting metastatic dissemination [12].

Within this framework, the SII, calculated as platelet count × neutrophil count / lymphocyte count, provides an integrated measure of tumor-promoting inflammation and impaired immune defense [13]. High SII values indicate an imbalance favoring pro-tumoral inflammation, immune suppression, and vascular dissemination. Although results across studies vary, multiple meta-analyses have consistently demonstrated that elevated SII correlates with reduced overall survival (OS) and PFS in several malignancies, including melanoma [14,15]. The biological rationale is consistent: elevated neutrophil and platelet counts amplify tumor-promoting cytokine signaling, while lymphopenia reflects weakened antitumor immunity [16]. Consequently, an increased SII represents a pro-inflammatory and immunosuppressive systemic state that fosters melanoma aggressiveness [17].

In the present study, an SII ≥560 was significantly associated with higher 12-month progression risk, with a relative risk of 5.3 and an odds ratio of 22.7. Kaplan-Meier analysis confirmed markedly divergent PFS outcomes-85% in patients with SII <560 versus 20% in those with SII ≥560. These findings support the prognostic relevance of SII not only in advanced stages, where aggressive behavior is expected, but also in early-stage melanoma that progresses rapidly despite favorable clinical classification. This suggests that SII captures biological aggressiveness mediated by systemic inflammation, not reflected in conventional staging parameters, and may have potential preoperative value in identifying high-risk patients before surgical excision.

Our findings are consistent with previous international evidence. Zhong et al. (2017), in a meta-analysis of 15 studies comprising 4,577 patients with various solid tumors, including, but not limited to, melanoma, demonstrated that elevated SII was associated with poorer OS (HR 1.55) and cancer-specific survival (HR 1.44), although heterogeneity in cutoff values and predominance of Asian cohorts were noted [2]. Similarly, Yang et al. (2018), analyzing 7,657 patients across 22 studies, confirmed the association between high SII and worse OS (HR 1.69) and PFS (HR 1.61) in multiple malignancies, again emphasizing the need for standardized thresholds [11]. More recently, Mesti et al. (2023) found that pretreatment SII independently predicted overall and PFS in metastatic melanoma patients treated with immunotherapy (HR 2.60 and HR 1.94, respectively), underscoring its utility even in advanced therapeutic settings [18].

Taken together, prior evidence and our results indicate that elevated SII serves as a robust and biologically plausible prognostic biomarker with potential preoperative and clinical utility in identifying patients at risk of early progression. Validation of the 560 cutoff, showing strong discriminative performance (AUC 0.91, sensitivity 84.2%, specificity 71.4%), supports its applicability across populations. Given its accessibility, reproducibility, and low cost, SII may represent a valuable adjunct to established prognostic models, particularly in settings with limited access to molecular or genomic assays.

The main limitations of this study include its retrospective, single-center design and modest sample size, which may limit external generalizability. Furthermore, the 12-month follow-up period may not capture late recurrences or long-term survival outcomes. Nonetheless, the internal consistency of results, biological plausibility, and concordance with prior literature reinforce the hypothesis that systemic inflammation plays a central role in melanoma progression. Future multicenter, prospective studies with longer follow-up and inclusion of additional variables such as ulceration, mitotic index, and treatment modality are warranted to validate these findings and refine the integration of SII into melanoma prognostic algorithms.

## Conclusions

In this retrospective cohort, the SII demonstrated strong prognostic value in melanoma, independently predicting 12-month progression even after adjustment for age and clinical stage. Elevated baseline SII (≥560) was significantly associated with reduced PFS, supporting its role as a simple, low-cost, and reproducible biomarker of biological aggressiveness. Notably, the index also identified early-stage cases (I-II) prone to rapid progression, suggesting that systemic inflammation may reflect tumor behavior beyond conventional staging. These findings highlight the potential of SII as a preoperative tool for risk stratification and personalized follow-up in melanoma. Future prospective multicenter studies with larger cohorts and extended follow-up are warranted to validate these results and to better define the clinical utility of SII as part of comprehensive melanoma risk stratification strategies.
